# Pachymeningeal Involvement with Blindness as the Presenting Manifestation of Non-Hodgkin Lymphoma

**DOI:** 10.4274/tjh.2016.0404

**Published:** 2018-03-06

**Authors:** Charanpreet Singh, Arjun Lakshman, Aditya Jandial, Sudha Sharma, Ram Nampoothiri, Gaurav Prakash, Pankaj Malhotra

**Affiliations:** 1 Postgraduate Institute of Medical Training and Research, Department of Internal Medicine, Chandigarh, India; 2 Postgraduate Institute of Medical Training and Research, Department of Internal Medicine, Clinical Hematology and Bone Marrow Division, Chandigarh, India; 3 Postgraduate Institute of Medical Training and Research, Department of Pathology, Chandigarh, India

**Keywords:** Non-Hodgkin lymphoma, Central nervous system involvement, Blindness, Papilledema

A 44-year-old female presented with fever for 6 months and gradual-onset progressive diminution of vision in both eyes for 1 month. On examination, she had enlarged cervical, axillary, and inguinal lymph nodes; hepatomegaly (7 cm under the right costal margin); splenomegaly (5 cm under the left costal margin); and bilateral renomegaly. Examination of the optic fundi (Figure 1A and 1B) showed bilateral disc edema (black arrowhead) with hemorrhages in the right eye (white arrowhead). Contrast-enhanced magnetic resonance imaging of the brain (Figure 2A) was done, which showed pachymeningeal enhancement (white arrow). Histopathological examination of the excised cervical lymph node showed infiltration by atypical lymphoid cells, with immunohistochemistry suggesting diffuse large B-cell lymphoma (DLBCL)-activated B-cell-like. Microscopic examination of cerebrospinal fluid showed infiltration by malignant lymphoid cells (Figure 2B). A diagnosis of non-Hodgkin lymphoma-DLBCL with secondary central nervous system (CNS) involvement and bilateral grade 4 papilledema, likely due to pachymeningeal involvement, was made. The patient was started on systemic and intrathecal chemotherapy.

CNS involvement with aggressive lymphomas is uncommon at initial presentation and usually occurs during relapse after primary therapy [1]. Ophthalmological abnormalities are usually attributed to the direct invasion of the optic nerve and ocular structures by the lymphoma [2], which was not seen in our case.

## Figures and Tables

**Figure 1:**
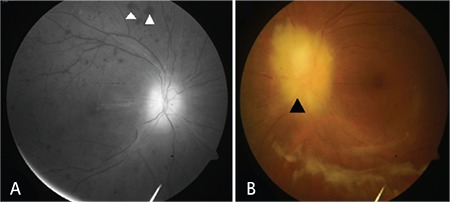
A) Right fundus photograph showing optic disc edema with multiple hemorrhages. B) Left fundus photograph showing large optic disc with blurred margins suggestive of papilledema.

**Figure 2:**
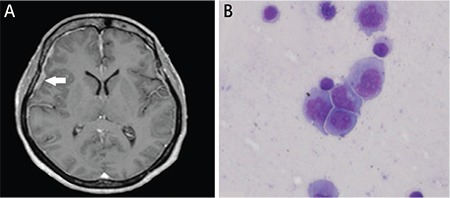
A) Contrast-enhanced magnetic resonance imaging of the brain showing patchy meningeal enhancement and thickening, suggestive of pachymeningitis. B) Cerebrospinal fluid cytology showing atypical lymphoid cells 2-3 times the size of normal lymphoid cells with prominent nucleoli.
